# Synchronous Sigmoid Colon and Small Bowel Adenocarcinomas: A Rare Case of Dual Primary Malignancies Presenting With Acute Bowel Obstruction

**DOI:** 10.7759/cureus.84877

**Published:** 2025-05-27

**Authors:** Beshr Mosa Basha, Shaher Abbarah, Yusuf kaaki, Naji Fliti

**Affiliations:** 1 General Surgery, Dr. Sulaiman Al Habib Medical Group, Khobar, SAU; 2 College of Medicine and Surgery, Almaarefa University, Riyadh, SAU; 3 General Surgery, Almoosa Specialist Hospital, Al Mubarraz, SAU

**Keywords:** adenocarcinoma, bowel obstruction, colorectal cancer, small bowel cancer, synchronous tumors

## Abstract

Synchronous colorectal and small bowel adenocarcinomas are exceptionally rare. This case report presents a 65-year-old male patient with acute bowel obstruction who underwent an emergency laparotomy. Intraoperative findings revealed synchronous sigmoid colon and small bowel tumors with peritoneal metastases. The patient underwent sigmoidectomy, small bowel resection, and metastasectomy. Histopathology confirmed two distinct primary adenocarcinomas. Postoperative management included KRAS mutation-guided chemotherapy. At the two-year follow-up, the patient remained disease-free. This case highlights the importance of thorough intraoperative exploration in emergency presentations and demonstrates the potential for favorable outcomes with aggressive multimodal treatment in synchronous gastrointestinal malignancies.

## Introduction

Synchronous tumors are defined as two or more primary neoplasms identified simultaneously or within a short time interval, with each tumor arising independently and not representing metastasis from the other. The International Association of Cancer Registries (IACR) and the International Agency for Research on Cancer (IARC) define synchronous multiple primary malignancies as two or more primary malignancies diagnosed within a six-month period [1].

Colorectal cancer (CRC) is among the most common malignancies globally, accounting for approximately 10% of all cancers and ranking as the second leading cause of cancer-related mortality [2]. In contrast, small bowel adenocarcinoma (SBA) is rare. Despite the small intestine constituting 75% of the length and 90% of the mucosal surface area of the gastrointestinal tract, SBA accounts for only 3%-5% of all gastrointestinal cancers, with the duodenum being the most commonly affected segment, followed by the jejunum and ileum [3].

Synchronous primary adenocarcinomas of the colon and small bowel are exceedingly rare. While synchronous CRCs occur in approximately 3.5% of CRC cases, the simultaneous presence of primary tumors in the colon and small intestine is far less common [4]. Due to its rarity, most data are derived from isolated case reports and small case series, particularly involving dual-site gastrointestinal adenocarcinomas, which often show a predilection for the jejunum and frequently present with intestinal obstruction [5].

## Case presentation

A 65-year-old male patient with type 2 diabetes mellitus presented to the emergency department with a five-day history of generalized abdominal pain and absolute constipation. His diabetes was well-managed with sitagliptin and metformin. Family history was unremarkable for malignancies. On examination, he appeared distressed, with a distended and tender abdomen. Lab investigation showed mild leukocytosis and an elevated CRP level (Table 1).

**Table 1:** Laboratory test results at presentation to the emergency department

Test	Result	Normal reference range
White blood cell (WBC)	13.80 x 10^9^/L	4.0-10.0 x 10^9^/L
Neutrophils (NEUT)	12.0 x 10^9^/L	2.0-7.5 x 10^9^/L
Neutrophil percentage (Neut%)	87.40%	40-75%
Hemoglobin (Hb)	15.5 g/dL	13.0-17.0 g/dL
Platelets (PLT)	215 x 10^9^/L	150-450 x 10^9^/L
C-reactive protein (CRP)	33 mg/L	<5 mg/L
Potassium (K+)	3.9 mmol/L	3.5-5.0 mmol/L
Sodium (Na+)	140 mmol/L	135-145 mmol/L
Alanine aminotransferase (ALT)	13 U/L	7-56 U/L
Aspartate aminotransferase (AST)	14 U/L	10-40 U/L
Gamma-glutamyl transferase (GGT)	22 U/L	9-48 U/L

**Figure 1:**
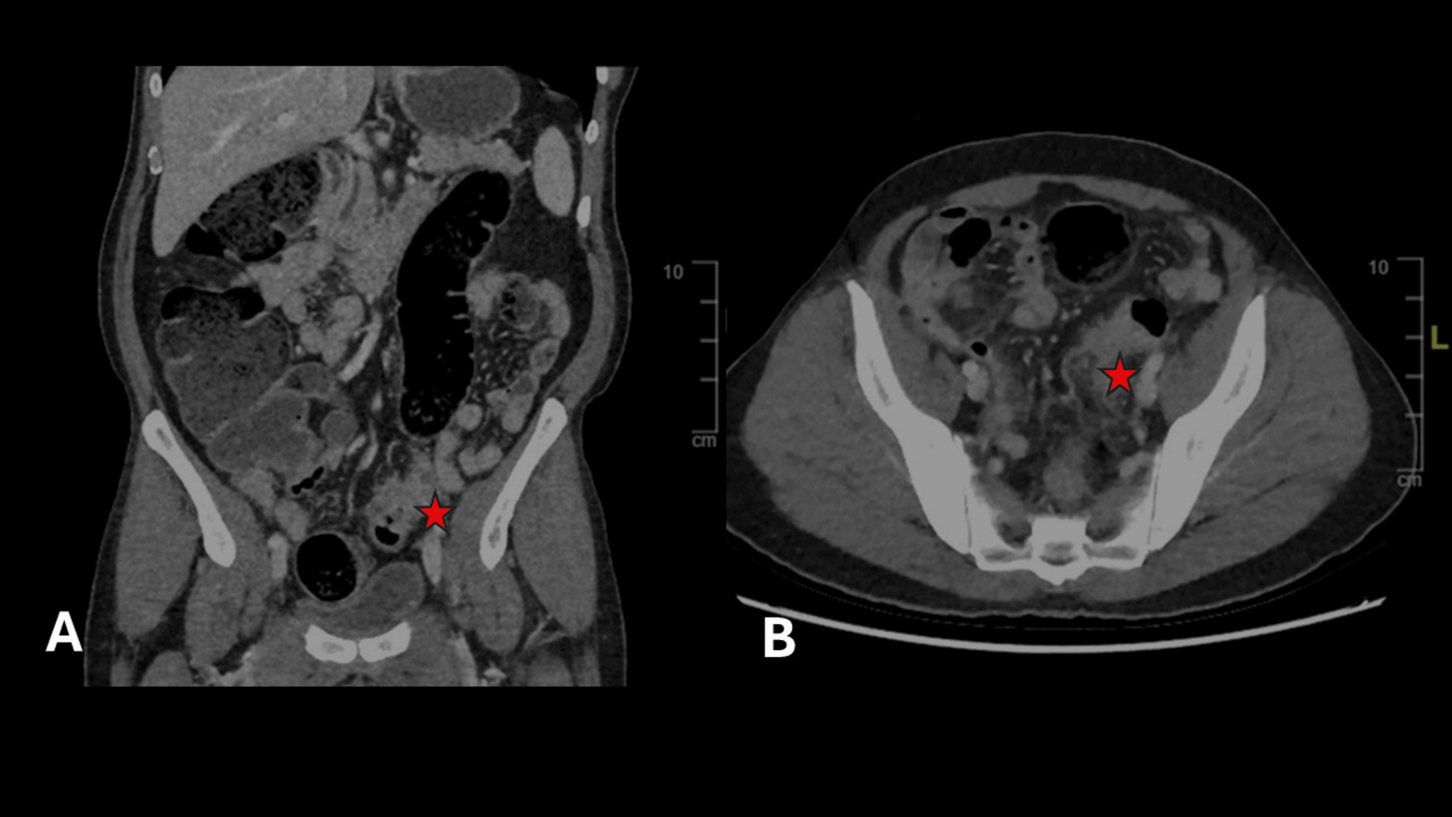
Abdominal CT showing sigmoid colon mass. (A) Coronal section. (B) Axial section

An abdominal X-ray revealed colonic dilation, fecal impaction, and multiple air-fluid levels. Contrast-enhanced CT of the abdomen and pelvis showed irregular circumferential wall thickening of the proximal sigmoid colon, causing significant luminal narrowing over a 2.5 cm segment (Figure 1). A milder, continuous thickening extended 7 cm distally, suggestive of malignant colonic obstruction.

**Figure 2:**
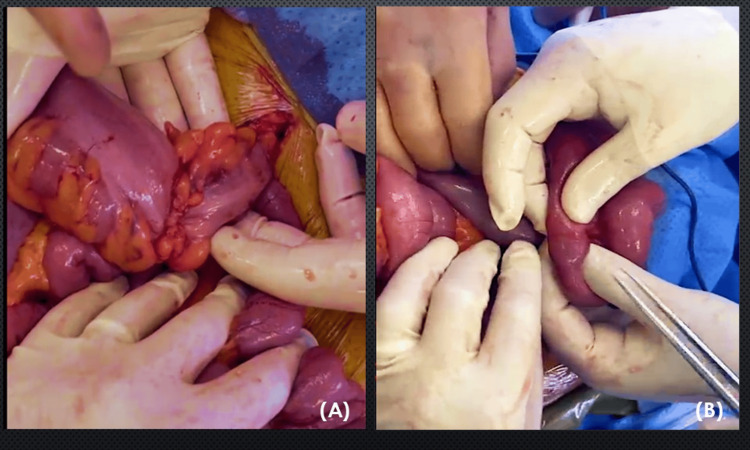
Intraoperative findings revealing tumor in the (A) sigmoid colon and (B) jejunum

**Figure 3:**
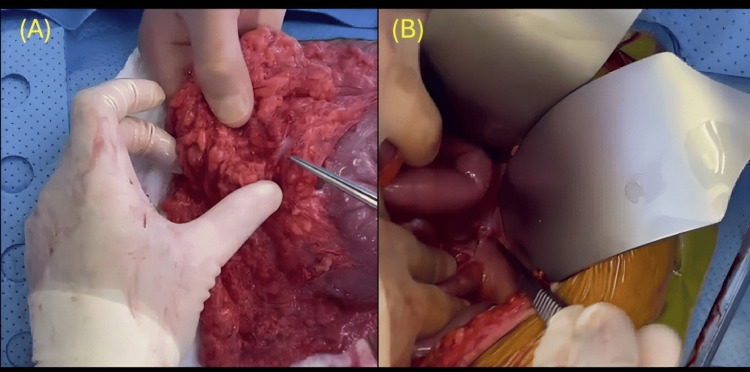
Intraoperative findings demonstrating tumor deposit in the (A) greater omentum and (B) peritoneum

Due to the acute presentation, the patient underwent an emergency exploratory laparotomy. Intraoperative findings included a sigmoid tumor (Figure 2A), ascitic fluid, and multiple omental (Figure 3A) and peritoneal (Figure 3B) metastases. Incidentally, a separate tumor was discovered in the jejunum (Figure 2B), approximately 40 cm from the ileocecal valve. Surgical procedures included sigmoidectomy with Hartmann’s pouch formation, small bowel resection with primary anastomosis, and debulking of visible peritoneal metastases.

**Figure 4:**
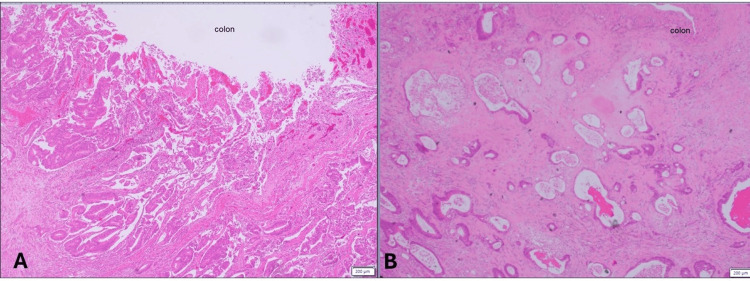
(A, B) Histopathological examination of the large bowel

**Figure 5:**
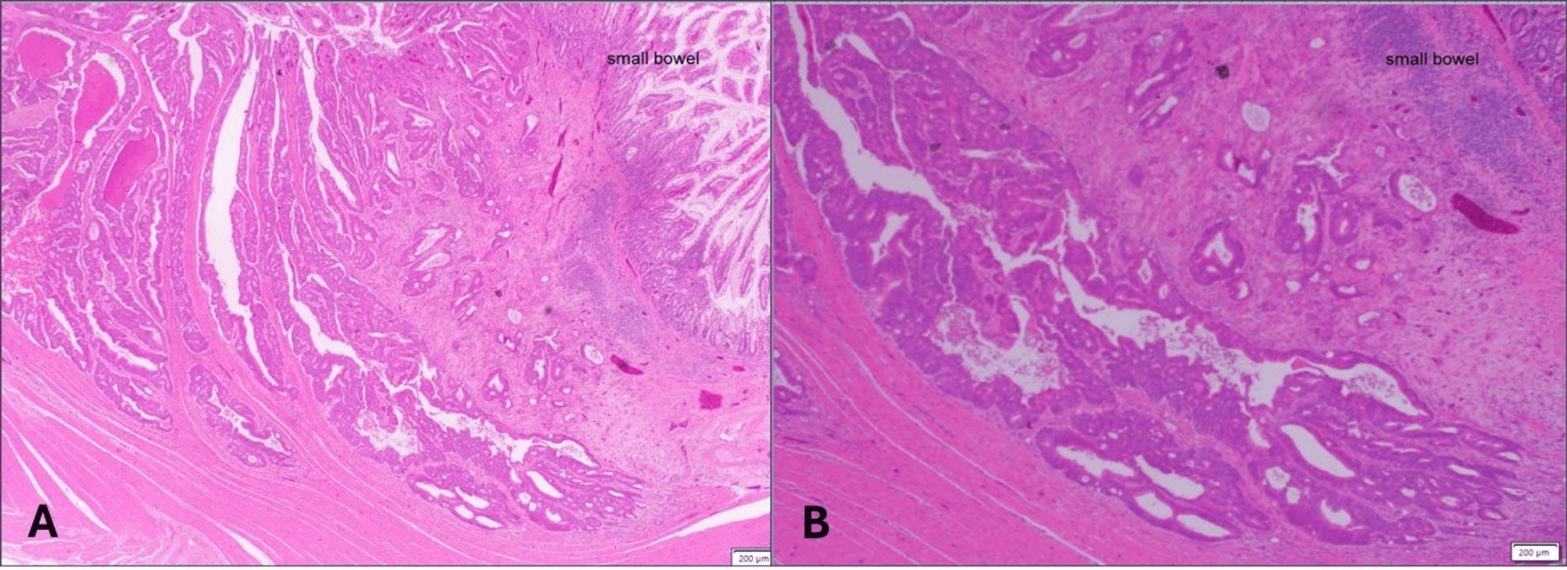
(A, B) Histopathological examination of the small bowel

Histopathological examination confirmed synchronous primary malignancies. The sigmoid tumor was a 3.0 cm moderately differentiated adenocarcinoma (pT4a N0 M1c) (Figure 4), with all 13 retrieved lymph nodes negative for metastasis. The jejunal mass measured 2.5 cm and was also a moderately differentiated adenocarcinoma (pT3 Nx) (Figure 5). Peritoneal implants were consistent with metastatic intestinal adenocarcinoma.

The postoperative course was uneventful. The patient was discharged on postoperative day 5 and referred to oncology. Molecular testing revealed a KRAS mutation. He subsequently received eight cycles of oxaliplatin and capecitabine (XELOX) chemotherapy. Follow-up colonoscopy at four months and PET/CT at five months showed no evidence of recurrence. Carcinoembryonic antigen (CEA) was 4.53 ng/mL one month postoperatively and remained within normal limits on all subsequent follow-up assessments. Colostomy reversal was performed at six months. At 36 months postoperatively, the patient remained disease-free.

## Discussion

The clinical presentation of SBA is typically nonspecific and may include vague abdominal pain, weight loss, gastrointestinal bleeding, or intermittent subacute obstruction [6]. However, in our patient, such symptoms were masked due to the dominant picture of large bowel obstruction caused by the sigmoid tumor. This obstructive presentation likely overshadowed or precluded the recognition of any subtle signs associated with the jejunal malignancy. The absence of classical SBA symptoms in our patient reinforces the importance of maintaining high suspicion for synchronous pathology, especially when intraoperative findings are unexpected. Synchronous tumors in the colon and small bowel are rare and may be missed preoperatively, especially if the bowel is significantly dilated, as was the case here. The sensitivity of standard contrast-enhanced abdominal CT in detecting SBA is modest, with an overall diagnostic accuracy of approximately 47% for its detection and staging [7]. In our case, the jejunal tumor was not detected preoperatively on abdominal CT. This may be attributed to the severely dilated bowel loops proximal to the obstructing sigmoid lesion, which likely resulted in improper luminal distension and poor visualization of the small bowel wall.

Intraoperative examination of the entire gastrointestinal tract is a critical component of CRC surgery, particularly in patients presenting with obstructing lesions, to avoid missing synchronous neoplasms. In our case, the incidental discovery of a jejunal adenocarcinoma during surgical exploration for a sigmoid obstruction reinforces this principle. While the literature predominantly discusses synchronous lesions within the colon and rectum, the underlying concept of meticulous intraoperative assessment remains universally applicable. Finan et al. emphasized the value of both colonoscopy and systematic intraoperative palpation in detecting synchronous colorectal carcinomas, noting that up to 60% of unexpected lesions were identified intraoperatively [8]. This underscores the necessity of thorough bowel examination, even beyond the colon, when managing obstructive colorectal tumors, as synchronous neoplasms, including those in the small bowel, may remain undetected on preoperative imaging.

From a genetic standpoint, synchronous tumors are often evaluated for hereditary syndromes such as Lynch syndrome, which is associated with a higher incidence of synchronous and metachronous colorectal and extracolonic tumors due to mismatch repair gene mutations [9]. Familial adenomatous polyposis (FAP) is a known hereditary risk factor associated with increased incidence of both small bowel and colorectal adenocarcinomas [6]. During postoperative follow-up, a colonoscopy revealed no evidence of FAP, suggesting the absence of a syndromic predisposition.

Standard management of CRC, particularly in stage III or metastatic disease, includes surgical resection followed by adjuvant chemotherapy [10]. Regimens such as XELOX or folinic acid, fluorouracil, and oxaliplatin (FOLFOX) are widely used and have demonstrated improved disease-free and overall survival. The addition of targeted therapy like bevacizumab further enhances outcomes in selected metastatic cases, especially in patients with KRAS wild-type tumors.

The prognosis of synchronous tumors varies depending on stage and response to treatment. Our patient demonstrated an excellent clinical course, having achieved complete remission following aggressive surgical resection and systemic chemotherapy. This outcome underscores the effectiveness of a combined approach and the importance of ongoing surveillance in managing such rare presentations [10].

## Conclusions

This rare case of synchronous colorectal and small bowel malignancies illustrates the diagnostic and therapeutic challenges of managing multifocal gastrointestinal cancers. A multidisciplinary approach combining surgical resection, molecular-guided adjuvant chemotherapy, and long-term surveillance facilitated favorable outcomes. This case highlights the need for enhanced diagnostic vigilance and personalized treatment strategies to improve the prognosis of patients with synchronous gastrointestinal neoplasms.
